# JAZF1: A metabolic actor subunit of the NuA4/TIP60 chromatin modifying complex

**DOI:** 10.3389/fcell.2023.1134268

**Published:** 2023-04-07

**Authors:** Amel Mameri, Jacques Côté

**Affiliations:** St. Patrick Research Group in Basic Oncology, Laval University Cancer Research Center, Oncology Division of CHU de Québec-Université Laval Research Center, Quebec City, QC, Canada

**Keywords:** epigenetics, acetylation, acetyl-CoA, gene expression, lipogenesis, lipolysis

## Abstract

The multisubunit NuA4/TIP60 complex is a lysine acetyltransferase, chromatin modifying factor and gene co-activator involved in diverse biological processes. The past decade has seen a growing appreciation for its role as a metabolic effector and modulator. However, molecular insights are scarce and often contradictory, underscoring the need for further mechanistic investigation. A particularly exciting route emerged with the recent identification of a novel subunit, JAZF1, which has been extensively linked to metabolic homeostasis. This review summarizes the major findings implicating NuA4/TIP60 in metabolism, especially in light of JAZF1 as part of the complex.

## Introduction

**FIGURE 1:**
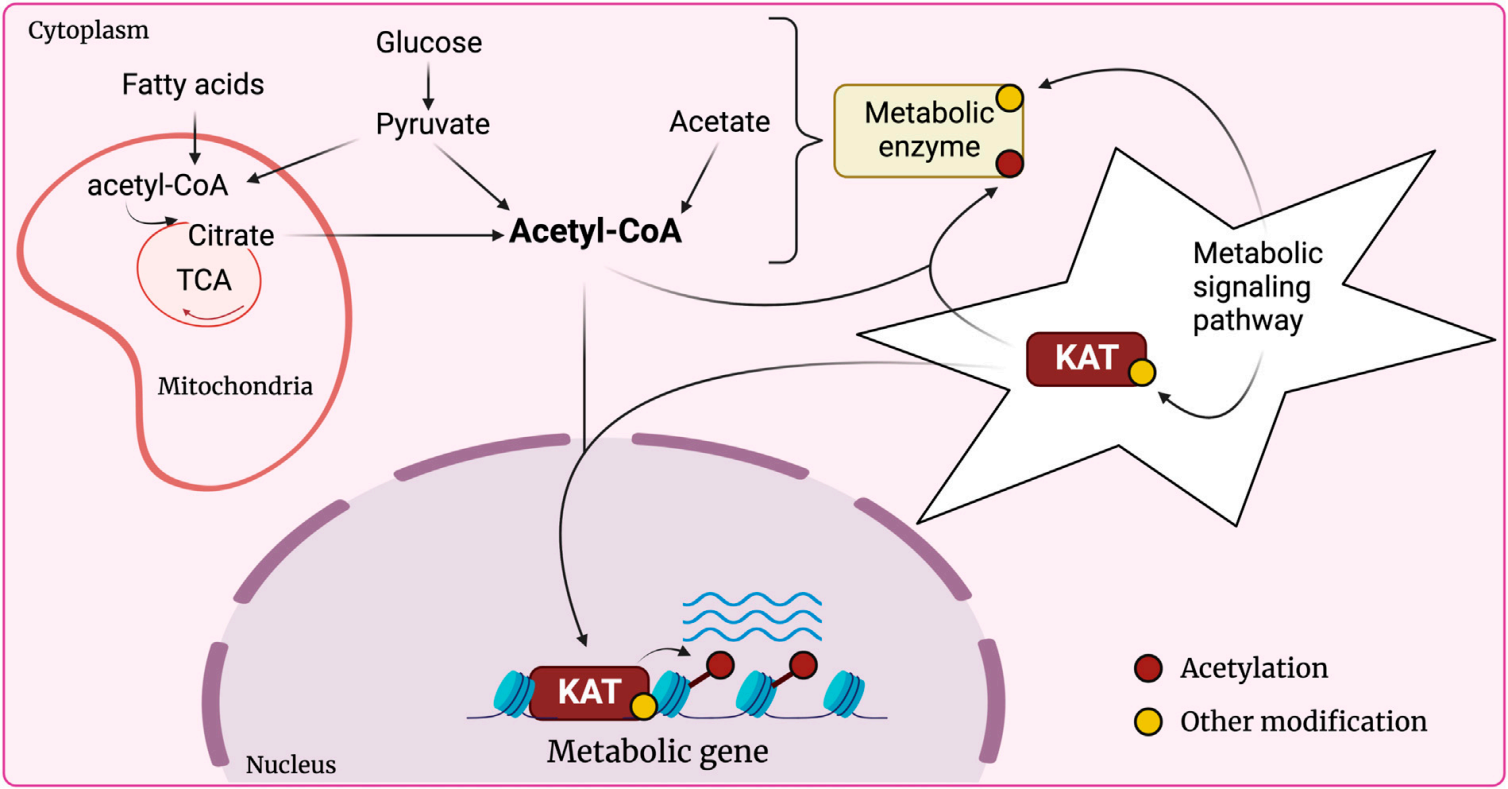
KATs at the nexus of crosstalk between chromatin modification and metabolism. Glucose and fatty acids constitute the main source of cytoplasmic acetyl-CoA, where pyruvate (product of glycolysis) and fatty acids enter the mitochondria to produce acetyl-CoA which feeds into the tricarboxylic acid (TCA) cycle and produces citrate. Citrate is then transported to the cytoplasm where it can yield acetyl-CoA. Other sources include acetate or direct conversion of cytoplasmic pyruvate into acetyl-CoA. The pool of acetyl-CoA available for KAT activity is sensitive to fluctuations in nutrient availability, activity of the metabolic enzymes that catalyze acetyl-CoA production, and the metabolic signaling cascades modulating metabolic enzymes. KATs can therefore communicate the metabolic state of the cell to chromatin and correspondingly adjust the transcriptional output, including that of metabolic genes. KATs themselves are involved in metabolic signaling as they are known to directly modify and regulate metabolic enzymes.

Lysine acetylation is a global regulatory post-translational modification catalyzed by lysine acetyltransferases (KATs). KATs are best characterized as modifiers of chromatin that hold special significance for cell metabolism. Chromatin, comprising genomic DNA and associated histones, is subject to a multitude of regulatory processes which allow for dynamic adjustment of transcriptional outputs in response to environmental cues. Histone acetylation is a mark of active genes that positively correlates with transcriptional activity. KATs utilize acetyl-CoA as a mandatory co-factor to acetylate their histone and non-histone substrates. Among other sources, glucose and lipid catabolism are the major contributors to cellular acetyl-CoA pools. Acetyl-CoA levels are therefore sensitive to the cellular metabolic context and can be limiting to KAT activity. It has thus been proposed that KATs function at the interface of crosstalk between metabolism and chromatin dynamics (Figure 1). Though metabolite availability unquestionably affects chromatin structure, and contrariwise, chromatin can alter metabolic processes at the transcriptional level, defining specific signaling crosstalk axes remains a challenge (Sheikh and Akhtar, 2019; Dai et al., 2020).

The multisubunit complex NuA4, also known as TIP60 in mammals, is an acetyltransferase that is structurally and functionally conserved across eukaryotes. Composed of 13 proteins in *Saccharomyces cerevisiae*, of which 12 have homologs in the 18-subunit human complex, including the catalytic subunit Tip60/Esa1 (Doyon et al., 2004; Altaf et al., 2009; Jacquet et al., 2016; Sudarshan et al., 2022) (Supplementary Table S1), NuA4/TIP60 is known to act as a co-activator at metabolic genes (Rossetto et al., 2014; Jacquet et al., 2016), as a direct modulator of metabolic enzymes (Lin et al., 2009; Rollins et al., 2017; Li et al., 2018; Cheng et al., 2019; Pham et al., 2022) and as an effector to metabolic signals that regulate autophagy (Lin et al., 2012; Yi et al., 2012; Cheng et al., 2019; Liu et al., 2021). Intriguingly, recent work has identified JAZF1 as a novel stable subunit of the complex (Piunti et al., 2019; Procida et al., 2021; Alerasool et al., 2022; Sudarshan et al., 2022). JAZF1 has been implicated in glucose and lipid homeostasis, insulin signaling, and metabolic disorders such as diabetes (Zeggini et al., 2008; Li et al., 2011; Ming et al., 2014b; Jang et al., 2014; Li et al., 2014; Yuan et al., 2015; Wei et al., 2018; Kobiita et al., 2020; Zhou et al., 2020; Ding et al., 2021; Ding et al., 2022). The growing appreciation for the importance of chromatin modifying KATs in metabolic processes necessitates revisiting the underappreciated role of NuA4/TIP60 in the metabolism-chromatin crosstalk network. In this review, we summarize key findings linking the NuA4/TIP60 complex to metabolism, with a special focus on its recently identified subunit JAZF1.

### JAZF1 strongly ties NuA4/TIP60 to metabolism

The human *JAZF1* locus is notoriously associated with type 2 diabetes (T2D). A gene variant defined by a single nucleotide polymorphism (SNP) mapping to intron 1 (rs864745-T) is the most widely reported to associate with T2D and was first identified as a significant T2D risk allele in a meta-analysis of genome-wide association studies (GWAS) conducted in individuals of European descent (Zeggini et al., 2008). Several population-based studies across ethnic groups have successfully replicated the finding (Omori et al., 2009; Rees et al., 2011; Cooke et al., 2012; Langberg et al., 2012; Ng et al., 2013; Chang et al., 2014; Song et al., 2015; Deng et al., 2017; García-Chapa et al., 2017). Other T2D-associated SNPs have also been identified on *JAZF1* (Sollis et al., 2022), many of which have additionally been linked to diabetes-relevant metabolic traits such as insulin secretion (Grarup et al., 2008; Boesgaard et al., 2009; Dai et al., 2022), insulin clearance (Goodarzi et al., 2013), plasma lipid levels (An et al., 2009; Dai et al., 2022) and body weight (Grarup et al., 2008; Kobiita et al., 2020; Zeng et al., 2021). Interestingly, all of the reported T2D risk SNPs on the *JAZF1* locus fall within a region of intron 1 thought to play a role in regulating *JAZF1* expression (Ding et al., 2022)*.* Particularly, the T2D risk allele rs1635852-T (van Hoek et al., 2008) exhibits *cis-*regulatory activity over *JAZF1* in pancreatic islets, resulting in reduced gene expression (Fogarty et al., 2013; Kobiita et al., 2020), while the rs864745-T allele correlates with lower *JAZF1* expression in skeletal muscle (Ding et al., 2022). These observations hint at the importance of adequate JAZF1 expression in maintaining a healthy metabolism and preventing metabolic disease. Indeed, JAZF1 is found downregulated in pancreatic beta-cells of diabetic patients (Marselli et al., 2010; Taneera et al., 2012; Kobiita et al., 2020), in liver cells of hepatic steatosis patients (Wei et al., 2018) and in metabolic tissues of diabetes and obesity rodent models (Li et al., 2014; Wei et al., 2018; Kobiita et al., 2020; Sághy et al., 2020; Zhou et al., 2020). Accordingly, *Jazf1* KO mice develop insulin resistance that is further exacerbated by high-fat diet feeding (Lee et al., 2022). In addition to genetic factors, there is evidence that the expression level of JAZF1 can be influenced by the metabolic context. Mice under a high-fat diet, mouse pancreatic islet cells cultured in high glucose and primary human islets cultured in a diabetogenic medium all show a decrease in JAZF1 mRNA and protein levels (Ng et al., 2013; Li et al., 2014; Wei et al., 2018; Kobiita et al., 2020).

To shed light on the metabolic function of JAZF1, several groups have investigated its activity in mammalian metabolic organs, including the pancreas, liver, adipose tissue, skeletal muscle and the hypothalamus. *In vivo* and *in vitro* studies alike argue for a pivotal role in the maintenance of glucose and lipid homeostasis, where JAZF1 enhances insulin signaling, promotes lipolysis and glucose uptake, and suppresses lipogenesis and gluconeogenesis in insulin-responsive tissues. Overexpression of JAZF1 upregulates the lipolytic enzymes HSL and ATGL and the glucose transporters GLUT1, GLUT2 and GLUT4, downregulates the lipogenic enzymes SREBP1, ACC1, FAS and HMGCR and the gluconeogenic enzymes PEPCK and G6Pase, and leads to increased phosphorylation of AMPK, IR, IRS-1 and Akt as well as increased insulin-stimulated PIP3 production, suggesting improved insulin sensitivity *via* the IR-PI3K-Akt pathway (Li et al., 2011; Ming et al., 2014b; Jang et al., 2014; Li et al., 2014; Yuan et al., 2015; Wei et al., 2018; Zhou et al., 2020; Ding et al., 2021; Ding et al., 2022). Consequently, JAZF1 overexpressing animals are protected against diet-induced insulin resistance and related metabolic dysfunctions (Jang et al., 2014; Li et al., 2014; Yuan et al., 2015; Wei et al., 2018; Zhou et al., 2020). Furthermore, JAZF1 has been linked to other equally important, metabolically relevant processes, including pancreatic beta cell differentiation and function (Taneera et al., 2012; Kobiita et al., 2020; Park et al., 2021), ribosome biogenesis (Kobiita et al., 2020; Procida et al., 2021) and adipogenesis (Ming et al., 2014a; Jeong et al., 2021).

**FIGURE 2:**
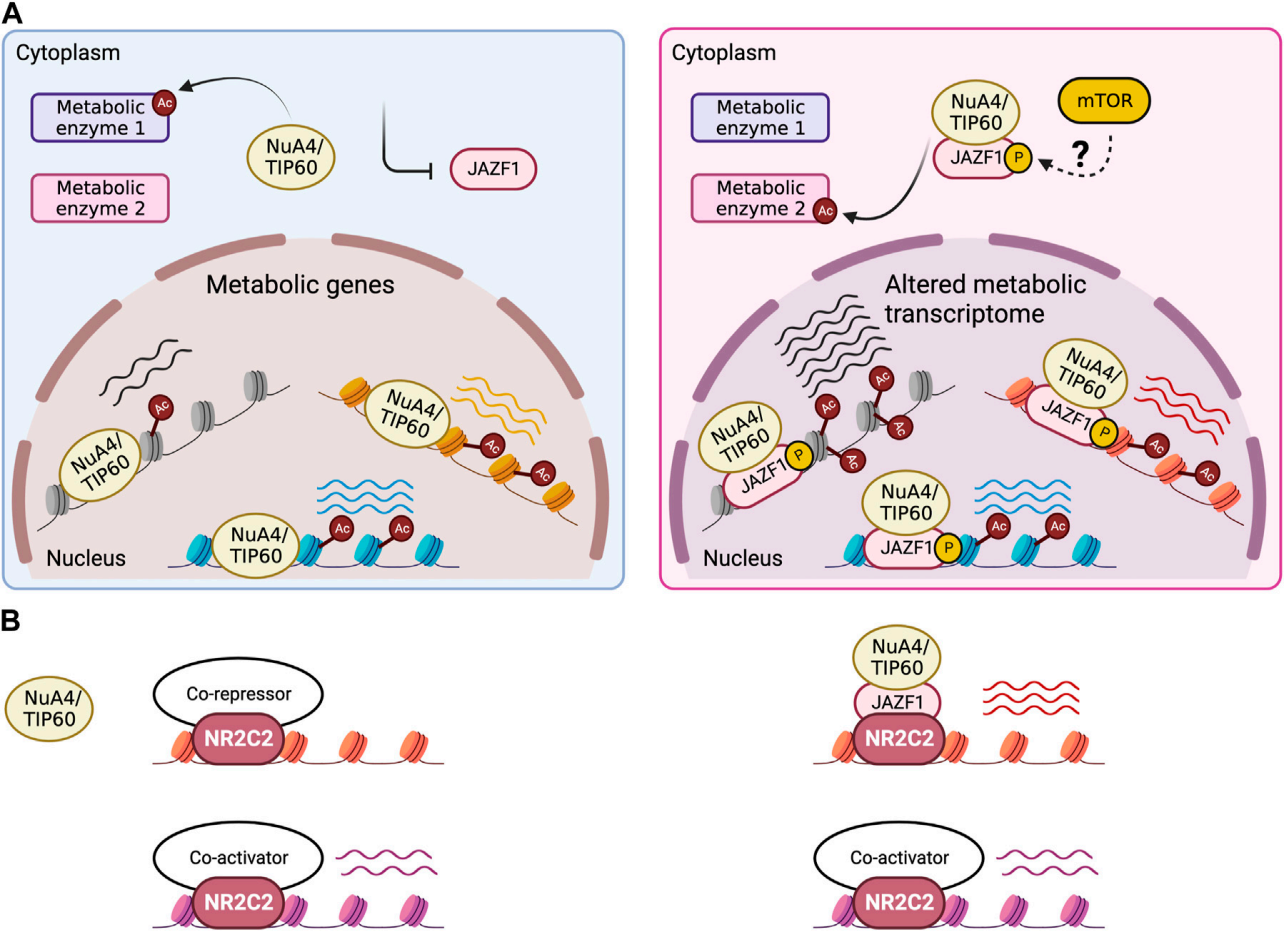
JAZF1 fine-tunes metabolic activity of the NuA4/TIP60 complex. Hypothetical model for the role of JAZF1 within the NuA4/TIP60 complex. **(A)** Enzymatic activity and specificity of the NuA4/TIP60 complex, either on chromatin at metabolic genes or towards metabolic enzymes, may be sensitive to the presence or absence of JAZF1 in the complex, which in turn could be controlled by expression, subcellular localization, or context-dependent assembly into the complex. Based on homology with Sfp1, mTOR-mediated phosphorylation may be involved in the process. **(B)** JAZF1 is possibly required for NR2C2-dependent recruitment of the NuA4/TIP60 complex at specific gene targets.

These valuable functional insights warrant a mechanistic dissection of the biochemical function of JAZF1, which is still lacking. Nevertheless, a few clues could be found in recent publications. JAZF1 was initially identified as a direct interactor of NR2C2, a nuclear hormone receptor and transcription factor with context-dependent activator or repressor function, where JAZF1 inhibited NR2C2 activity on a reporter plasmid without affecting its homodimerization or DNA binding efficiency. It was therefore suggested that JAZF1 was a co-repressor of NR2C2 (Nakajima, 2004). However, most of the literature characterizing JAZF1 at the biochemical level argues for a co-activator function. JAZF1 has been reported to associate with the NuA4/TIP60 transcription co-activator complex in different human cell lines (Piunti et al., 2019; Procida et al., 2021; Alerasool et al., 2022). Using a reliable approach for the purification and characterization of native protein complexes, consisting of tandem affinity purification of the target protein at physiological expression levels followed by proteomic analysis (Dalvai et al., 2015), our group has established JAZF1 as a stable stoichiometric subunit of NuA4/TIP60 (Sudarshan et al., 2022). Moreover, a chromosomal translocation frequently occurring in a type of sarcoma which merges JAZF1 and SUZ12, a subunit of the co-repressor methyltransferase complex PRC2, mistargets the acetyltransferase activity of NuA4/TIP60 to PRC2-occupied loci, leading to their upregulation (Sudarshan et al., 2022; Tavares et al., 2022). In addition, JAZF1 seems to positively regulate acetylation of the histone H2A.Z at intronic enhancers, a chromatin modification that is specifically deposited by Tip60 (Procida et al., 2021). Remarkably, JAZF1 was detected as a top hit in a dCas9-based screen for transcription activators and co-activators in human cells (Alerasool et al., 2022). While we detected NR2C2 in our tandem affinity based-proteomic analysis of JAZF1 interactome, it was clearly sub-stoichiometric relative to NuA4/TIP60 subunits with similar molecular weights (Sudarshan et al., 2022). This suggests that the complex might be acting as a co-activator of NR2C2, with JAZF1 mediating the interaction. In fact, genome-wide NR2C2 binding sites in different human cell lines are enriched in RNA processing and protein translation genes (O'Geen et al., 2010), similar to JAZF1 (Kobiita et al., 2020; Tavares et al., 2022) and other NuA4/TIP60 subunits (Jacquet et al., 2016). The same study also found that NR2C2 exhibited cell type-specific binding at genes involved in lipid and carbohydrate metabolism, and that most of NR2C2 target loci are active genes (O'Geen et al., 2010). Overall, the current literature is in favor of a model whereby JAZF1 carries out its metabolic functions as an integral component of the acetyltransferase complex NuA4/TIP60, with possible cooperation with the transcription factor NR2C2 (Figure 2).

### A link to the TOR growth pathway in lower eukaryotes

In *S. cerevisiae*, the transcription factor Sfp1 bears significant homology with mammalian JAZF1 (Kobiita et al., 2020; Sudarshan et al., 2022) (Supplementary Table S1). Sfp1 is best known for its critical role in modulating yeast growth, where it tightly controls the growth rate and positively regulates cell size at division (Jorgensen et al., 2002; Cipollina et al., 2008; Fazio et al., 2008). Mechanistically, Sfp1 achieves this through its function as a major transcriptional activator of protein biosynthesis genes, including ribosome biogenesis, ribosomal protein and other translation-related genes (Jorgensen et al., 2002; Fingerman et al., 2003; Cipollina et al., 2008; Fazio et al., 2008; Albert et al., 2019; Zencir et al., 2020). Crucially, there is mounting evidence of a potential interaction between Sfp1 and yeast NuA4. An interactome analysis found that a subunit of NuA4, Tra1, associates with Sfp1 (Lempiäinen et al., 2009), and an acetylome analysis detected NuA4-dependent acetylation of Sfp1 (Downey et al., 2015). However, unlike JAZF1, Sfp1 does not seem to be a stable subunit of the NuA4 complex, considering the low recovery of NuA4 subunits in the interactome analysis (Lempiäinen et al., 2009). Thus, the biochemical nature of interaction appears to be different in yeast. Nonetheless, as in human, the yeast NuA4 complex occupies ribosomal protein and ribosome biogenesis genes (Rossetto et al., 2014), suggesting that Sfp1 and NuA4 co-regulate expression at these loci and arguing for some functional conservation relative to higher eukaryotes.

Sfp1 activity is highly sensitive to metabolic cues. For instance, glucose stimulates translocation of Sfp1 from the cytoplasm into the nucleus and leads to Sfp1-mediated gene activation (Marion et al., 2004; Cipollina et al., 2008; Albert et al., 2019). Interestingly, this nuclear-cytoplasmic shuttling of Sfp1, the main mechanism for regulation of its activity, is governed by the kinase TORC1, a major metabolic signaling hub (Marion et al., 2004; Lempiäinen et al., 2009; Zencir et al., 2020). TORC1 directly phosphorylates Sfp1, resulting in its localization to the nucleus and subsequent binding and upregulation of target genes (Lempiäinen et al., 2009). This raises the question of whether JAZF1 could similarly be modified and modulated by TORC1 in mammals (Figure 2A).

Aside from protein synthesis, Sfp1 has been reported to positively regulate glycolysis *via* upregulation of the glycolytic gene *HXK2* (Cipollina et al., 2008), implying a role in glucose homeostasis, and to negatively regulate lipid droplet formation *via* downregulation of the lipogenic gene *DGA1* (Teixeira et al., 2021), suggesting a role in lipid homeostasis, all reminiscent of functional data on JAZF1. Curiously, Sfp1 inhibits TORC1-mediated phosphorylation of Sch9, the closest yeast homolog to the mammalian Akt proteins (Supplementary Table S1). This seems to be a negative feedback loop, as the two act in parallel pathways downstream of TORC1 to induce ribosome biogenesis and ribosomal protein gene activation (Jorgensen et al., 2002; Jorgensen et al., 2004; Lempiäinen et al., 2009).

### Beyond JAZF1: Other metabolic functions of the NuA4/TIP60 complex

Both mammalian and yeast NuA4/TIP60 have been linked to the same metabolic processes described for JAZF1 and Sfp1, either *via* the catalytic acetyltransferase subunit or other proteins in the complex (Supplementary Figure S1). A CRISPR knock-out screen in mouse pancreatic beta cells identified seven NuA4/TIP60 subunits (Tip60, ING3, DMAP1, p400, BRD8, BAF53A and MRGBP) as positive regulators of insulin secretion, involving the complex in beta cell function and arguing for a potential role in diabetes (Fang et al., 2019). Moreover, mammalian Tip60 and BRD8 are essential for adipogenesis and together with p400 are recruited as co-activators by the key adipogenic transcription factor PPARγ (van Beekum et al., 2008; Grönniger et al., 2010; Couture et al., 2012). Additionally, enzymatic activity of PCK1, the rate-limiting enzyme in gluconeogenesis, is positively controlled by Esa1/Tip60-mediated acetylation in yeast and human, supporting a role in glucose homeostasis (Lin et al., 2009). In parallel, disruption of yeast NuA4 leads to increased glycogen biosynthesis through direct regulation of protein kinase A (Filteau et al., 2015; Walden et al., 2020). Further, implication of the NuA4/TIP60 complex in lipid homeostasis is clear, albeit somewhat controversial. In *S. cerevisiae*, NuA4 subunits Esa1, Eaf1 and Eaf7 promote fatty acid biosynthesis and lipid droplet formation by enhancing activity of the fatty acid biosynthesis enzyme ACC1 (Dacquay et al., 2017; Rollins et al., 2017; Pham et al., 2022). In mammals, there is evidence for Tip60 functioning in the opposing processes of lipogenesis and lipolysis. Tip60-mediated Lipin-1 acetylation is an activating modification which triggers Lipin-1 translocation to the ER where it catalyzes a critical step in triacylglyceride synthesis. This process is likely conserved in yeast (Li et al., 2018). Yet, Tip60-mediated acetylation of Pacer, an important effector in the process of autophagy, negatively regulates lipid droplet accumulation (Cheng et al., 2019). In parallel, during glucose deprivation, Tip60 promotes lipolysis of lipid droplets through direct acetylation of choline kinase (CHK) alpha2 (Liu et al., 2021). Finally, Tip60 suppresses the PI3K-Akt pathway in some types of cancer (Yang et al., 2017; Zhang et al., 2018), while Esa1 suppresses the equivalent pathway by acetylating Sip2 (homolog of the mammalian AMPKβ) which promotes its interaction with Snf1 (homolog of the mammalian AMPKα) and inhibition of Snf1-mediated phosphorylation of Sch9 (homolog of Akt) (Lu et al., 2011) (Supplementary Table S1).

## Discussion

In light of the current literature, JAZF1 has emerged as a critical modulator of cell metabolism in health and disease. While the functional relationship between JAZF1 and metabolic homeostasis is well-established, the underlying mechanisms require further investigation. An exciting research avenue would be to define the specific role of JAZF1 within the multisubunit acetyltransferase complex NuA4/TIP60. As a newly identified subunit of NuA4/TIP60 (Sudarshan et al., 2022), it is unclear if or how JAZF1 regulates enzymatic activity of the complex on or off chromatin. It is especially interesting that the role of NuA4/TIP60 activity in transcription of metabolic genes and direct regulation of metabolic enzymes is well supported (van Beekum et al., 2008; Lin et al., 2009; Grönniger et al., 2010; Couture et al., 2012; Rossetto et al., 2014; Filteau et al., 2015; Jacquet et al., 2016; Dacquay et al., 2017; Rollins et al., 2017; Li et al., 2018; Cheng et al., 2019; Walden et al., 2020; Liu et al., 2021; Pham et al., 2022). Therefore, it is tempting to speculate that JAZF1 specifically modulates metabolism-related activity of the NuA4/TIP60 complex.

Although JAZF1 and the rest of the NuA4/TIP60 complex are essentially implicated in the same metabolic processes, a few contradictions are noted and need to be addressed. First, while JAZF1 suppresses gluconeogenesis (Yuan et al., 2015; Zhou et al., 2020), NuA4/TIP60 is known to promote it (Lin et al., 2009). Second, JAZF1 has a pro-lipolytic anti-lipogenic function (Li et al., 2011; Ming et al., 2014b; Jang et al., 2014; Li et al., 2014; Wei et al., 2018), whereas NuA4/Tip60 was found to positively regulate either lipolysis or lipogenesis in different studies (Dacquay et al., 2017; Rollins et al., 2017; Li et al., 2018; Cheng et al., 2019; Wei et al., 2020; Liu et al., 2021; Pham et al., 2022). Lastly, JAZF1 stimulates the PI3K-Akt pathway downstream of insulin signaling (Yuan et al., 2015; Zhou et al., 2020), but NuA4/TIP60 was found to suppress it (Lu et al., 2011; Yang et al., 2017; Zhang et al., 2018). These conflicting observations could be reconciled by considering a potential role for JAZF1 in fine-tuning metabolic activity of the NuA4/TIP60 complex, an idea that is supported by several lines of evidence. Expression level of JAZF1 appears to be tightly controlled in response to environmental metabolites (Ng et al., 2013; Li et al., 2014; Wei et al., 2018; Kobiita et al., 2020). This suggests that there might be metabolic contexts in which the NuA4/TIP60 complex exists in the cell without JAZF1. Furthermore, subcellular localization could be another regulatory layer. JAZF1 is mainly classified as a nuclear protein (www.proteinatlas.org; (Uhlen et al., 2015)) but work in mouse primary beta cells has shown glucose-dependent shuttling from the cytoplasm to the nucleus (Kobiita et al., 2020), implying the possibility that at least in some mammalian tissues the mechanism described for yeast Sfp1 is conserved. Together, the control of JAZF1 expression and/or nuclear localization in response to metabolic cues may converge to dictate its temporal association with the NuA4/TIP60 complex, which in turn would favor some metabolic functions of the complex over others (Figure 2A). Since JAZF1 directly interacts with NR2C2, a transcription factor targeting metabolic genes (Nakajima, 2004; O'Geen et al., 2010), it would be interesting to test if JAZF1 is required for NR2C2-dependent recruitment of NuA4/TIP60 to certain metabolic genes (Figure 2B), or if JAZF1 mediates the interaction to facilitate NuA4/TIP60-dependent acetylation of NR2C2 in order to modulate its transcriptional program. The discovery of JAZF1 as a *bona fide* subunit of the NuA4/TIP60 complex calls for different methodological approaches aimed at delineating its metabolic function than what has been applied in the past. Future work may thus unravel previously unknown molecular processes underpinning the role of JAZF1 in maintaining metabolic homeostasis. In turn, this can also lead to novel therapeutic approaches targeting JAZF1 association with NuA4/TIP60 to treat specific metabolic disorders.

## References

[B1] Albert B. Tomassetti S. Gloor Y. Dilg D. Mattarocci S. Kubik S. (2019). Sfp1 regulates transcriptional networks driving cell growth and division through multiple promoter-binding modes. Genes & Dev. 33, 288–293. 10.1101/gad.322040.118 30804227 PMC6411004

[B2] Alerasool N. Leng H. Lin Z.-Y. Gingras A.-C. Taipale M. (2022). Identification and functional characterization of transcriptional activators in human cells. Mol. Cell 82, 677–695.e7. 10.1016/j.molcel.2021.12.008 35016035

[B3] Altaf M. Auger A. Covic M. Cote J. (2009). Connection between histone H2A variants and chromatin remodeling complexes. Biochem. Cell Biol. 87, 35–50. 10.1139/O08-140 19234522

[B4] An P. Feitosa M. Ketkar S. Adelman A. Lin S. Borecki I. (2009). Epistatic interactions of CDKN2B-TCF7L2 for risk of type 2 diabetes and of CDKN2B-JAZF1 for triglyceride/high-density lipoprotein ratio longitudinal change: Evidence from the framingham heart study. BMC Proc. 3 (7), S71. 10.1186/1753-6561-3-s7-s71 20018066 PMC2795973

[B5] Boesgaard T. W. Gjesing A. P. Grarup N. Rutanen J. Jansson P.-A. Hribal M. L. (2009). Variant near ADAMTS9 known to associate with type 2 diabetes is related to insulin resistance in offspring of type 2 diabetes patients--EUGENE2 study. PloS One 4, e7236. 10.1371/journal.pone.0007236 19789630 PMC2747270

[B6] Chang Y.-C. Liu P.-H. Yu Y.-H. Kuo S.-S. Chang T.-J. Jiang Y.-D. (2014). Validation of type 2 diabetes risk variants identified by genome-wide association studies in han Chinese population: A replication study and meta-analysis. PloS One 9, e95045. 10.1371/journal.pone.0095045 24736664 PMC3988150

[B7] Cheng X. Ma X. Zhu Q. Song D. Ding X. Li L. (2019). Pacer is a mediator of mTORC1 and GSK3-TIP60 signaling in regulation of autophagosome maturation and lipid metabolism. Mol. Cell 73, 788–802.e7. 10.1016/j.molcel.2018.12.017 30704899

[B8] Cipollina C. Van Den Brink J. Daran-Lapujade P. Pronk J. T. Vai M. De Winde J. H. (2008). Revisiting the role of yeast Sfp1 in ribosome biogenesis and cell size control: A chemostat study. Microbiol. Read. Engl. 154, 337–346. 10.1099/mic.0.2007/011767-0 18174152

[B9] Cooke J. N. Ng M. C. Y. Palmer N. D. An S. S. Hester J. M. Freedman B. I. (2012). Genetic risk assessment of type 2 diabetes-associated polymorphisms in African Americans. Diabetes Care 35, 287–292. 10.2337/dc11-0957 22275441 PMC3263882

[B10] Couture J.-P. Nolet G. Beaulieu E. Blouin R. Gévry N. (2012). The p400/Brd8 chromatin remodeling complex promotes adipogenesis by incorporating histone variant H2A.Z at PPARγ target genes. Endocrinology 153, 5796–5808. 10.1210/en.2012-1380 23064015

[B11] Dacquay L. Flint A. Butcher J. Salem D. Kennedy M. Kaern M. (2017). NuA4 lysine acetyltransferase complex contributes to phospholipid homeostasis in *Saccharomyces cerevisiae* . G3 (Bethesda, Md.) 7, 1799–1809. 10.1534/g3.117.041053 28455416 PMC5473759

[B12] Dai H. Qian Y. Lv H. Jiang L. Jiang H. Shen M. (2022). Rs864745 in JAZF1, an islet function associated variant, correlates with plasma lipid levels in both type 1 and type 2 diabetes status, but not healthy subjects. Front. Endocrinol. 13, 898893. 10.3389/fendo.2022.898893 PMC9283698 35846288

[B13] Dai Z. Ramesh V. Locasale J. W. (2020). The evolving metabolic landscape of chromatin biology and epigenetics. Nat. Rev. Genet. 21, 737–753. 10.1038/s41576-020-0270-8 32908249 PMC8059378

[B14] Dalvai M. Loehr J. Jacquet K. Huard C. C. Roques C. Herst P. (2015). A scalable genome-editing-based approach for mapping multiprotein complexes in human cells. Cell Rep. 13, 621–633. 10.1016/j.celrep.2015.09.009 26456817

[B15] Deng X. Liu H. Nalima N. Qiqiger A. Zhu J. (2017). Association of polymorphisms rs290487, rs864745, rs4430796 and rs23136 with type 2 diabetes in the Uyghur population in China. Int. J. Clin. Exp. Pathology 10, 8813–8819. PMC6965464 31966747

[B16] Ding Q. Zhao W. Long J. Alsafar H. Zhou Q. Chen H. (2022). Cis-regulation of antisense non-coding RNA at the JAZF1 locus in type 2 diabetes. J. Gene Med. 24, e3407. 10.1002/jgm.3407 34978128

[B17] Ding Z. Sun D. Han J. Shen L. Yang F. Sah S. (2021). Novel noncoding RNA CircPTK2 regulates lipolysis and adipogenesis in cachexia. Mol. Metab. 53, 101310. 10.1016/j.molmet.2021.101310 34311131 PMC8365522

[B18] Downey M. Johnson J. R. Davey N. E. Newton B. W. Johnson T. L. Galaang S. (2015). Acetylome profiling reveals overlap in the regulation of diverse processes by sirtuins, gcn5, and esa1. Mol. Cell. proteomics MCP 14, 162–176. 10.1074/mcp.M114.043141 25381059 PMC4288252

[B19] Doyon Y. Selleck W. Lane W. S. Tan S. Côté J. (2004). Structural and functional conservation of the NuA4 histone acetyltransferase complex from yeast to humans. Mol. Cell. Biol. 24, 1884–1896. 10.1128/MCB.24.5.1884-1896.2004 14966270 PMC350560

[B20] Fang Z. Weng C. Li H. Tao R. Mai W. Liu X. (2019). Single-cell heterogeneity analysis and CRISPR screen identify key β-cell-specific disease genes. Cell Rep. 26, 3132–3144.e7. 10.1016/j.celrep.2019.02.043 30865899 PMC6573026

[B21] Fazio A. Jewett M. C. Daran-Lapujade P. Mustacchi R. Usaite R. Pronk J. T. (2008). Transcription factor control of growth rate dependent genes in *Saccharomyces cerevisiae*: A three factor design. BMC genomics 9, 341. 10.1186/1471-2164-9-341 18638364 PMC2500033

[B22] Filteau M. Diss G. Torres-Quiroz F. Dube A. K. Schraffl A. Bachmann V. A. (2015). Systematic identification of signal integration by protein kinase A. Proc. Natl. Acad. Sci. U. S. A. 112, 4501–4506. 10.1073/pnas.1409938112 25831502 PMC4394313

[B23] Fingerman I. Nagaraj V. Norris D. Vershon A. K. (2003). Sfp1 plays a key role in yeast ribosome biogenesis. Eukaryot. Cell 2, 1061–1068. 10.1128/EC.2.5.1061-1068.2003 14555489 PMC219361

[B24] Fogarty M. P. Panhuis T. M. Vadlamudi S. Buchkovich M. L. Mohlke K. L. (2013). Allele-specific transcriptional activity at type 2 diabetes-associated single nucleotide polymorphisms in regions of pancreatic islet open chromatin at the JAZF1 locus. Diabetes 62, 1756–1762. 10.2337/db12-0972 23328127 PMC3636602

[B25] García-Chapa E. G. Leal-Ugarte E. Peralta-Leal V. Durán-González J. Meza-Espinoza J. P. (2017). Genetic epidemiology of type 2 diabetes in Mexican mestizos. BioMed Res. Int. 2017, 3937893. 10.1155/2017/3937893 28607931 PMC5451767

[B26] Goodarzi M. O. Guo X. Cui J. Jones M. R. Haritunians T. Xiang A. H. (2013). Systematic evaluation of validated type 2 diabetes and glycaemic trait loci for association with insulin clearance. Diabetologia 56, 1282–1290. 10.1007/s00125-013-2880-6 23494448 PMC3651757

[B27] Grarup N. Andersen G. Krarup N. T. Albrechtsen A. Schmitz O. Jørgensen T. (2008). Association testing of novel type 2 diabetes risk alleles in the JAZF1, CDC123/CAMK1D, TSPAN8, THADA, ADAMTS9, and NOTCH2 loci with insulin release, insulin sensitivity, and obesity in a population-based sample of 4,516 glucose-tolerant middle-aged Danes. Diabetes 57, 2534–2540. 10.2337/db08-0436 18567820 PMC2518507

[B28] Grönniger E. Wessel S. Kühn S. C. Söhle J. Wenck H. Stäb F. (2010). A new protocol for functional analysis of adipogenesis using reverse transfection technology and time-lapse video microscopy. Cell Biol. Int. 34, 737–746. 10.1042/CBI20090299 20359292

[B29] Jacquet K. Fradet-Turcotte A. Avvakumov N. Lambert J. P. Roques C. Pandita R. K. (2016). The TIP60 complex regulates bivalent chromatin recognition by 53BP1 through direct H4K20me binding and H2AK15 acetylation. Mol. Cell 62, 409–421. 10.1016/j.molcel.2016.03.031 27153538 PMC4887106

[B30] Jang W. Y. Bae K. B. Kim S. H. Yu D. H. Kim H. J. Ji Y. R. (2014). Overexpression of Jazf1 reduces body weight gain and regulates lipid metabolism in high fat diet. Biochem. Biophysical Res. Commun. 444, 296–301. 10.1016/j.bbrc.2013.12.094 24380856

[B31] Jeong J. Jang S. Park S. Kwon W. Kim S.-Y. Jang S. (2021). JAZF1 heterozygous knockout mice show altered adipose development and metabolism. Cell & Biosci. 11, 161. 10.1186/s13578-021-00625-1 PMC8375039 34407873

[B32] Jorgensen P. Nishikawa J. L. Breitkreutz B.-J. Tyers M. (2002). Systematic identification of pathways that couple cell growth and division in yeast. Sci. (New York, N.Y.) 297, 395–400. 10.1126/science.1070850 12089449

[B33] Jorgensen P. Rupes I. Sharom J. R. Schneper L. Broach J. R. Tyers M. (2004). A dynamic transcriptional network communicates growth potential to ribosome synthesis and critical cell size. Genes & Dev. 18, 2491–2505. 10.1101/gad.1228804 15466158 PMC529537

[B34] Kobiita A. Godbersen S. Araldi E. Ghoshdastider U. Schmid M. W. Spinas G. (2020). The diabetes gene JAZF1 is essential for the homeostatic control of ribosome biogenesis and function in metabolic stress. Cell Rep. 32, 107846. 10.1016/j.celrep.2020.107846 32640216

[B35] Langberg K. A. Ma L. Sharma N. K. Hanis C. L. Elbein S. C. Hasstedt S. J. (2012). Single nucleotide polymorphisms in JAZF1 and BCL11A gene are nominally associated with type 2 diabetes in African-American families from the GENNID study. J. Hum. Genet. 57, 57–61. 10.1038/jhg.2011.133 22113416 PMC3266455

[B36] Lee H. Y. Jang H. R. Li H. Samuel V. T. Dudek K. D. Osipovich A. B. (2022). Deletion of Jazf1 gene causes early growth retardation and insulin resistance in mice. Proc. Natl. Acad. Sci. U. S. A. 119, e2213628119. 10.1073/pnas.2213628119 36442127 PMC9894197

[B37] Lempiäinen H. Uotila A. Urban J. Dohnal I. Ammerer G. Loewith R. (2009). Sfp1 interaction with TORC1 and Mrs6 reveals feedback regulation on TOR signaling. Mol. Cell 33, 704–716. 10.1016/j.molcel.2009.01.034 19328065

[B38] Li L. Yang Y. Yang G. Lu C. Yang M. Liu H. (2011). The role of JAZF1 on lipid metabolism and related genes *in vitro* . Metabolism Clin. Exp. 60, 523–530. 10.1016/j.metabol.2010.04.021 20580384

[B39] Li T. Y. Song L. Sun Y. Li J. Yi C. Lam S. M. (2018). Tip60-mediated lipin 1 acetylation and ER translocation determine triacylglycerol synthesis rate. Nat. Commun. 9, 1916. 10.1038/s41467-018-04363-w 29765047 PMC5953937

[B40] Li X. Yang M. Wang H. Jia Y. Yan P. Boden G. (2014). Overexpression of JAZF1 protected ApoE-deficient mice from atherosclerosis by inhibiting hepatic cholesterol synthesis via CREB-dependent mechanisms. Int. J. Cardiol. 177, 100–110. 10.1016/j.ijcard.2014.09.007 25499349

[B41] Lin S. Y. Li T. Y. Liu Q. Zhang C. Li X. Chen Y. (2012). GSK3-TIP60-ULK1 signaling pathway links growth factor deprivation to autophagy. Science 336, 477–481. 10.1126/science.1217032 22539723

[B42] Lin Y.-Y. Lu J.-Y. Zhang J. Walter W. Dang W. Wan J. (2009). Protein acetylation microarray reveals that NuA4 controls key metabolic target regulating gluconeogenesis. Cell 136, 1073–1084. 10.1016/j.cell.2009.01.033 19303850 PMC2696288

[B43] Liu R. Lee J. H. Li J. Yu R. Tan L. Xia Y. (2021). Choline kinase alpha 2 acts as a protein kinase to promote lipolysis of lipid droplets. Mol. Cell 81, 2722–2735.e9. 10.1016/j.molcel.2021.05.005 34077757

[B44] Lu J.-Y. Lin Y.-Y. Sheu J.-C. Wu J.-T. Lee F.-J. Chen Y. (2011). Acetylation of yeast AMPK controls intrinsic aging independently of caloric restriction. Cell 146, 969–979. 10.1016/j.cell.2011.07.044 21906795 PMC3176974

[B45] Marion R. M. Regev A. Segal E. Barash Y. Koller D. Friedman N. (2004). Sfp1 is a stress- and nutrient-sensitive regulator of ribosomal protein gene expression. Proc. Natl. Acad. Sci. U. S. A. 101, 14315–14322. 10.1073/pnas.0405353101 15353587 PMC521938

[B46] Marselli L. Thorne J. Dahiya S. Sgroi D. C. Sharma A. Bonner-Weir S. (2010). Gene expression profiles of Beta-cell enriched tissue obtained by laser capture microdissection from subjects with type 2 diabetes. PloS One 5, e11499. 10.1371/journal.pone.0011499 20644627 PMC2903480

[B47] Ming G.-F. Li X. Yin J.-Y. Ai Y.-H. Xu D.-M. Ma X.-H. (2014a). JAZF1 regulates visfatin expression in adipocytes via PPARα and PPARβ/δ signaling. Metabolism Clin. Exp. 63, 1012–1021. 10.1016/j.metabol.2014.05.006 24930994

[B48] Ming G.-F. Xiao D. Gong W.-J. Liu H.-X. Liu J. Zhou H.-H. (2014b). JAZF1 can regulate the expression of lipid metabolic genes and inhibit lipid accumulation in adipocytes. Biochem. Biophysical Res. Commun. 445, 673–680. 10.1016/j.bbrc.2014.02.088 24583129

[B49] Nakajima T. Fujino S. Nakanishi G. Kim Y. S. Jetten A. M. (2004). TIP27: A novel repressor of the nuclear orphan receptor TAK1/TR4. Nucleic Acids Res. 32, 4194–4204. 10.1093/nar/gkh741 15302918 PMC514368

[B50] Ng M. C. Y. Saxena R. Li J. Palmer N. D. Dimitrov L. Xu J. (2013). Transferability and fine mapping of type 2 diabetes loci in african Americans: The candidate gene association resource plus study. Diabetes 62, 965–976. 10.2337/db12-0266 23193183 PMC3581206

[B51] O'geen H. Lin Y.-H. Xu X. Echipare L. Komashko V. M. He D. (2010). Genome-wide binding of the orphan nuclear receptor TR4 suggests its general role in fundamental biological processes. BMC Genomics 11, 689. 10.1186/1471-2164-11-689 21126370 PMC3019231

[B52] Omori S. Tanaka Y. Horikoshi M. Takahashi A. Hara K. Hirose H. (2009). Replication study for the association of new meta-analysis-derived risk loci with susceptibility to type 2 diabetes in 6,244 Japanese individuals. Diabetologia 52, 1554–1560. 10.1007/s00125-009-1397-5 19455301

[B53] Park S. J. Kwon W. Park S. Jeong J. Kim D. Jang S. (2021). Jazf1 acts as a regulator of insulin-producing β-cell differentiation in induced pluripotent stem cells and glucose homeostasis in mice. FEBS J. 288, 4412–4427. 10.1111/febs.15751 33555104

[B54] Pham T. Walden E. Huard S. Pezacki J. Fullerton M. D. Baetz K. (2022). Fine-tuning acetyl-CoA carboxylase 1 activity through localization: Functional genomics reveals a role for the lysine acetyltransferase NuA4 and sphingolipid metabolism in regulating Acc1 activity and localization. Genetics 221, iyac086. 10.1093/genetics/iyac086 35608294 PMC9339284

[B55] Piunti A. Smith E. R. Morgan M. a. J. Ugarenko M. Khaltyan N. Helmin K. A. (2019). Catacomb: An endogenous inducible gene that antagonizes H3K27 methylation activity of Polycomb repressive complex 2 via an H3K27M-like mechanism. Sci. Adv. 5, eaax2887. 10.1126/sciadv.aax2887 31281901 PMC6609211

[B56] Procida T. Friedrich T. Jack A. P. M. Peritore M. Bönisch C. Eberl H. C. (2021). JAZF1, A novel p400/TIP60/NuA4 complex member, regulates H2A.Z acetylation at regulatory regions. Int. J. Mol. Sci. 22, 678. 10.3390/ijms22020678 33445503 PMC7826843

[B57] Rees S. D. Hydrie M. Z. I. Shera A. S. Kumar S. O'hare J. P. Barnett A. H. (2011). Replication of 13 genome-wide association (GWA)-validated risk variants for type 2 diabetes in Pakistani populations. Diabetologia 54, 1368–1374. 10.1007/s00125-011-2063-2 21350842

[B58] Rollins M. Huard S. Morettin A. Takuski J. Pham T. T. Fullerton M. D. (2017). Lysine acetyltransferase NuA4 and acetyl-CoA regulate glucose-deprived stress granule formation in *Saccharomyces cerevisiae* . PLoS Genet. 13, e1006626. 10.1371/journal.pgen.1006626 28231279 PMC5344529

[B59] Rossetto D. Cramet M. Wang A. Y. Steunou A. L. Lacoste N. Schulze J. M. (2014). Eaf5/7/3 form a functionally independent NuA4 submodule linked to RNA polymerase II-coupled nucleosome recycling. EMBO J. 33, 1397–1415. 10.15252/embj.201386433 24843044 PMC4194127

[B60] Sághy É. Vörös I. Ágg B. Kiss B. Koncsos G. Varga Z. V. (2020). Cardiac miRNA expression and their mRNA targets in a rat model of prediabetes. Int. J. Mol. Sci. 21, E2128. 10.3390/ijms21062128 PMC7139428 32244869

[B61] Sheikh B. N. Akhtar A. (2019). The many lives of KATs — Detectors, integrators and modulators of the cellular environment. Nat. Rev. Genet. 20, 7–23. 10.1038/s41576-018-0072-4 30390049

[B62] Sollis E. Mosaku A. Abid A. Buniello A. Cerezo M. Gil L. (2022). The NHGRI-EBI GWAS catalog: Knowledgebase and deposition resource. Nucleic Acids Res. 51, D977–D985. 10.1093/nar/gkac1010 PMC9825413 36350656

[B63] Song M. Zhao F. Ran L. Dolikun M. Wu L. Ge S. (2015). The Uyghur population and genetic susceptibility to type 2 diabetes: Potential role for variants in CDKAL1, JAZF1, and IGF1 genes. Omics A J. Integr. Biol. 19, 230–237. 10.1089/omi.2014.0162 PMC4390191 25785549

[B64] Sudarshan D. Avvakumov N. Lalonde M.-E. Alerasool N. Joly-Beauparlant C. Jacquet K. (2022). Recurrent chromosomal translocations in sarcomas create a megacomplex that mislocalizes NuA4/TIP60 to Polycomb target loci. Genes & Dev. 36, 664–683. 10.1101/gad.348982.121 35710139 PMC9296003

[B65] Taneera J. Lang S. Sharma A. Fadista J. Zhou Y. Ahlqvist E. (2012). A systems genetics approach identifies genes and pathways for type 2 diabetes in human islets. Cell Metab. 16, 122–134. 10.1016/j.cmet.2012.06.006 22768844

[B66] Tavares M. Khandelwal G. Muter J. Viiri K. Beltran M. Brosens J. J. (2022). JAZF1-SUZ12 dysregulates PRC2 function and gene expression during cell differentiation. Cell Rep. 39, 110889. 10.1016/j.celrep.2022.110889 35649353 PMC9637993

[B67] Teixeira V. Martins T. S. Prinz W. A. Costa V. (2021). Target of rapamycin complex 1 (TORC1), protein kinase A (pka) and cytosolic pH regulate a transcriptional circuit for lipid droplet formation. Int. J. Mol. Sci. 22, 9017. 10.3390/ijms22169017 34445723 PMC8396576

[B68] Uhlen M. Fagerberg L. Hallstrom B. M. Lindskog C. Oksvold P. Mardinoglu A. (2015). Proteomics. Tissue-based map of the human proteome. Science 347, 1260419. 10.1126/science.1260419 25613900

[B69] Van Beekum O. Brenkman A. B. Grøntved L. Hamers N. Van Den Broek N. J. F. Berger R. (2008). The adipogenic acetyltransferase Tip60 targets activation function 1 of peroxisome proliferator-activated receptor gamma. Endocrinology 149, 1840–1849. 10.1210/en.2007-0977 18096664

[B70] Van Hoek M. Dehghan A. Witteman J. C. M. Van Duijn C. M. Uitterlinden A. G. Oostra B. A. (2008). Predicting type 2 diabetes based on polymorphisms from genome-wide association studies: A population-based study. Diabetes 57, 3122–3128. 10.2337/db08-0425 18694974 PMC2570410

[B71] Walden E. A. Fong R. Y. Pham T. T. Knill H. Laframboise S. J. Huard S. (2020). Phenomic screen identifies a role for the yeast lysine acetyltransferase NuA4 in the control of Bcy1 subcellular localization, glycogen biosynthesis, and mitochondrial morphology. PLoS Genet. 16, e1009220. 10.1371/journal.pgen.1009220 33253187 PMC7728387

[B72] Wei Q. Zhou B. Yang G. Hu W. Zhang L. Liu R. (2018). JAZF1 ameliorates age and diet-associated hepatic steatosis through SREBP-1c -dependent mechanism. Cell Death Dis. 9, 859. 10.1038/s41419-018-0923-0 30154417 PMC6113258

[B73] Wei Y. Tian C. Zhao Y. Liu X. Liu F. Li S. (2020). MRG15 orchestrates rhythmic epigenomic remodelling and controls hepatic lipid metabolism. Nat. Metab. 2, 447–460. 10.1038/s42255-020-0203-z 32694659

[B74] Yang Y. Sun J. Chen T. Tao Z. Zhang X. Tian F. (2017). Tat-interactive protein-60kda (TIP60) regulates the tumorigenesis of lung cancer *in vitro* . J. Cancer 8, 2277–2281. 10.7150/jca.19677 28819431 PMC5560146

[B75] Yi C. Ma M. Ran L. Zheng J. Tong J. Zhu J. (2012). Function and molecular mechanism of acetylation in autophagy regulation. Science 336, 474–477. 10.1126/science.1216990 22539722

[B76] Yuan L. Luo X. Zeng M. Zhang Y. Yang M. Zhang L. (2015). Transcription factor TIP27 regulates glucose homeostasis and insulin sensitivity in a PI3-kinase/Akt-dependent manner in mice. Int. J. Obes. 39, 949–958. 10.1038/ijo.2015.5 25614086

[B77] Zeggini E. Scott L. J. Saxena R. Voight B. F. Marchini J. L. Hu T. (2008). Meta-analysis of genome-wide association data and large-scale replication identifies additional susceptibility loci for type 2 diabetes. Nat. Genet. 40, 638–645. 10.1038/ng.120 18372903 PMC2672416

[B78] Zencir S. Dilg D. Rueda M. P. Shore D. Albert B. (2020). Mechanisms coordinating ribosomal protein gene transcription in response to stress. Nucleic Acids Res. 48, 11408–11420. 10.1093/nar/gkaa852 33084907 PMC7672434

[B79] Zeng Y. He H. Zhang L. Zhu W. Shen H. Yan Y.-J. (2021). GWA-based pleiotropic analysis identified potential SNPs and genes related to type 2 diabetes and obesity. J. Hum. Genet. 66, 297–306. 10.1038/s10038-020-00843-4 32948839 PMC7884093

[B80] Zhang Y. Ji G. Han S. Shao Z. Lu Z. Huo L. (2018). Tip60 suppresses cholangiocarcinoma proliferation and metastasis via PI3k-AKT. Cell. Physiology Biochem. 50, 612–628. 10.1159/000494183 30308494

[B81] Zhou M. Xu X. Wang H. Yang G. Yang M. Zhao X. (2020). Effect of central JAZF1 on glucose production is regulated by the PI3K-Akt-AMPK pathway. FASEB J. official Publ. Fed. Am. Soc. Exp. Biol. 34, 7058–7074. 10.1096/fj.201901836RR 32275331

