# Supplementary material for: JAZF1: A metabolic actor subunit of the NuA4/TIP60 chromatin modifying complex
Source: Front Cell Dev Biol. 2023 Apr 7;11:1134268. doi: 10.3389/fcell.2023.1134268 (PMC10119425; doi:10.3389/fcell.2023.1134268)
Supplement: Supplementary file 1 [file Table1.pdf]

<u>Supplementary Table S1.</u> Homology between subunits of the yeast and the human NuA4/TIP60 complexes and other proteins involved in the metabolic pathways regulated by them.

	Saccharomyces cerevisiae	***Homo sapiens***	
**NuA4**	Tra1	TRRAP	**NuA4/TIP60**
	Eaf1	p400(EP400)	
	Epl1	EPC1/EPC2	
	Swc4	DMAP1	
	Arp4	BAF53A(ACTL6A)	
	Esa1(KAT5)	Tip60(KAT5)	
	Eaf3	MRG15/MRGX(MORF4L1/2)	
	Act1	Actin	
	Yng2	ING3	
	Yaf9	GAS41(YEATS4)	
	Eaf6	hEAF6(MEAF6)	
	Eaf7	MRGBP	
	Eaf5		
		BRD8	
		YL-1(VPS72)	
		RUVBL1	
		RUVBL2	
		MBTD1	
	Sfp1	JAZF1	
**SNF1**	Snf1	AMPKα	**AMPK**
	Sip2/Sip1/Gal38	AMPKß	
	Snf4	AMPKγ	
	Sch9	Akt1/Akt2/Akt3	
