# Supplementary material for: JAZF1: A metabolic actor subunit of the NuA4/TIP60 chromatin modifying complex
Source: Front Cell Dev Biol. 2023 Apr 7;11:1134268. doi: 10.3389/fcell.2023.1134268 (PMC10119425; doi:10.3389/fcell.2023.1134268)

<u>Supplementary Figure S1.</u> Metabolic processes involving the NuA4/TIP60 complex.

Summary of the metabolic pathways modulated by subunits of the NuA4/TIP60 complex in yeast and mammals.

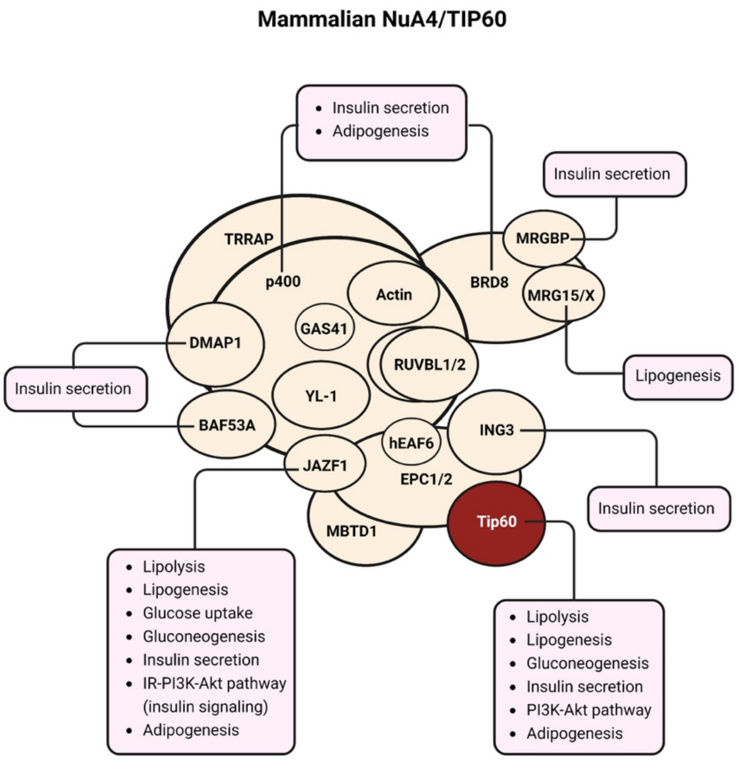

Supplement: Supplementary file 2 [file Presentation1.pdf]
